# Coherent Raman scattering microscopy: capable solution in search of a larger audience

**DOI:** 10.1117/1.JBO.26.6.060601

**Published:** 2021-06-03

**Authors:** Richard C. Prince, Eric O. Potma

**Affiliations:** a University of California, Irvine, Department of Biomedical Engineering, Irvine, California, United States; b University of California, Irvine, Department of Chemistry, Irvine, California, United States

**Keywords:** coherent Raman scattering microscopy, optical imaging, lipid metabolism

## Abstract

**Significance:** Coherent Raman scattering (CRS) microscopy is an optical imaging technique with capabilities that could benefit a broad range of biomedical research studies.

**Aim:** We reflect on the birth, rapid rise, and inescapable growing pains of the technique and look back on nearly four decades of developments to examine where the CRS imaging approach might be headed in the next decade to come.

**Approach:** We provide a brief historical account of CRS microscopy, followed by a discussion of the challenges to disseminate the technique to a larger audience. We then highlight recent progress in expanding the capabilities of the CRS microscope and assess its current appeal as a practical imaging tool.

**Results:** New developments in Raman tagging have improved the specificity and sensitivity of the CRS technique. In addition, technical advances have led to CRS microscopes that can capture hyperspectral data cubes at practical acquisition times. These improvements have broadened the application space of the technique.

**Conclusion:** The technical performance of the CRS microscope has improved dramatically since its inception, but these advances have not yet translated into a substantial user base beyond a strong core of enthusiasts. Nonetheless, new developments are poised to move the unique capabilities of the technique into the hands of more users.

## 1 New Imaging Technique is Born

**Fig. 1:**
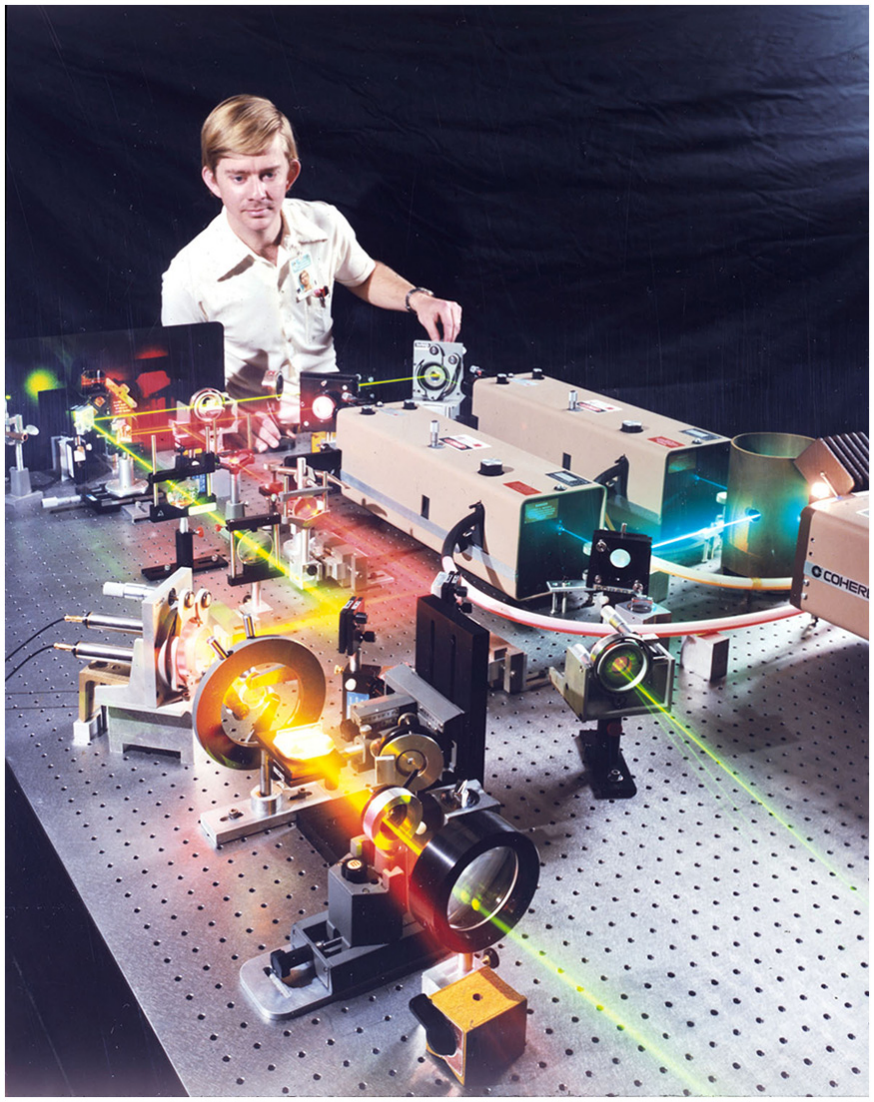
Dr. Michael Duncan adjusts the world’s first coherent Raman microscope built at Naval Research Laboratory in 1981. Courtesy of Michael Duncan.

The benefits and applications of new optical imaging approaches are not always evident at the inception of the technique. The first coherent Raman scattering (CRS) microscope, built in 1981 and shown in Fig. 1, was an unmistakable technological advance. The system produced images of onion cells in heavy water^1^ as well as deuterated multi-lamellar vesicles.^2^ At the same time, the immediate impact of the device remained largely unclear. Although it was recognized that the CRS signal levels could “exceed those produced in spontaneous Raman processes”^3^ and that spurious fluorescence contributions could be largely avoided, an application space in which the attributes of CRS microscopy made a clear difference stayed out of sight.

Even after substantial improvements to the CRS microscope were reported in 1999,^4^ experts in the field of optical microscopy continued to be skeptical about the prospects of the still-nascent CRS technology.^5^ Such skepticism was not surprising since images required up to 30 min to record. Yet, less than a decade later, further developments had improved the imaging rate to such an extent that speed became CRS microscopy’s most celebrated asset.^6^^–^^9^ It was now possible to create time-lapses of dynamic processes and image 3D tissue volumes with rates comparable to fluorescence.

The speed of CRS imaging allowed the study of water dynamics in cells and tissues.^10^^–^^13^ And the exceptional contrast generated by lipids led to visualization of lipids in cultured cells, tissue slices, and live animals. The high CRS signals obtained from pooled lipid in, otherwise, aqueous environments enabled a wide range of studies that uncovered various aspects of lipid biology,^14^ with applications in the areas of intracellular lipid metabolism,^15^^–^^17^ atherosclerosis,^18^^–^^20^ neurodegenerative diseases,^21^^–^^25^ the role of lipids in cancer biology,^26^^–^^28^ and many more. Thanks to the technique’s successes in lipid imaging, its reputation as a biological and biomedical imaging technique grew. On the other hand, these successes also cemented the notion that CRS microscopy is synonymous with lipid mapping and not much else.

## 2 Growing Pains and a Renewed Focus

**Fig. 2:**
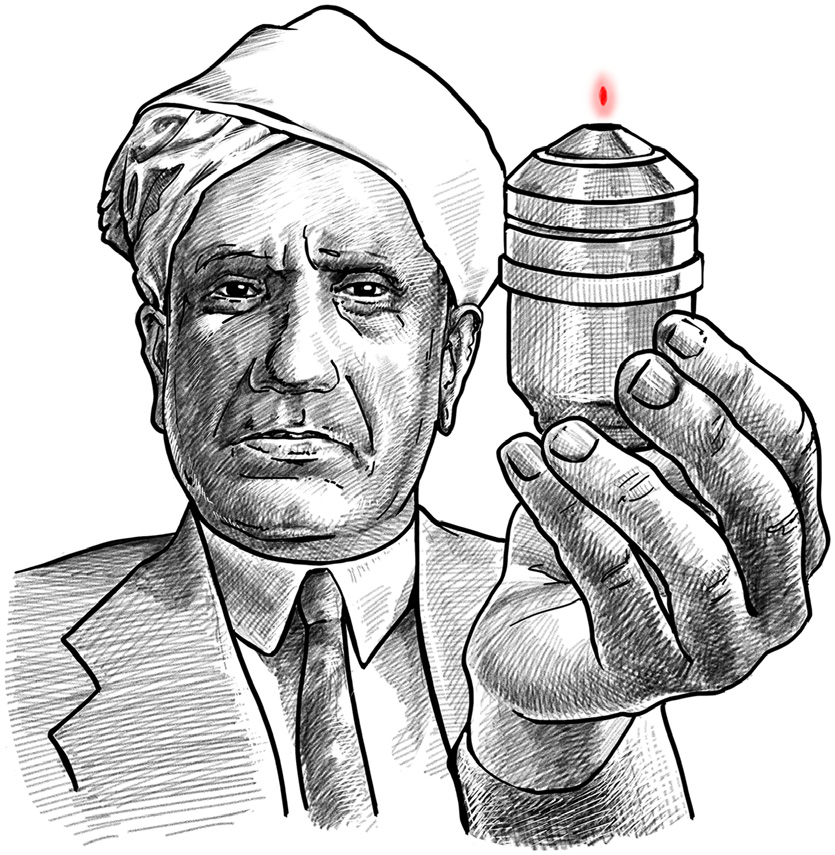
Raman-based imaging, here symbolized by C. V. Raman holding a high numerical aperture lens, combines the molecular selectivity of the Raman effect with the high resolution of optical imaging (drawing by Potma). Coherent Raman imaging has significantly improved the speed of the Raman-based imaging, opening up the technique to a myriad of biomedical imaging applications.

The Raman effect senses molecules through their characteristic vibrational modes. Using this capability in a microscope, symbolized in Fig. 2, makes it possible to generate chemically selective images without the use of labels. The CRS microscope builds on these properties but improves on a key weakness of Raman microscopy, namely its unfavorable imaging speed. The much higher image acquisition rate offered through the nonlinear CRS mechanism implies that the Raman effect can now be used for label-free mapping of live cells and tissues—applications for which fast imaging capabilities are of essence.

Despite CRS’s highly desirable imaging properties, the adoption of the technique by the biomedical imaging community has been a slow and seemingly protracted process. Primarily driven by a core group of devoted developers, CRS methods began making inroads into university imaging facilities, neuroscience laboratories, and clinical settings. By the end of the 2000s, there appeared to be sufficient impetus for commercializing the CRS microscopy technique. The first CRS imaging system to hit the market was produced by Olympus, followed several years later by a dedicated coherent anti-Stokes Raman scattering (CARS) microscope by Leica Microsystems. The hope was that CRS microscopy would expand to other areas of biological imaging and that the technique would claim its place as a routine tool for biological research. Despite impressive studies that showed the possibility of CRS to map a variety of biochemical compounds beyond lipids, the method did not easily outgrow its reputation as a lipid-imaging tool. Widespread adoption was lacking, with many instruments existing only in large optical development labs. The high cost, complexity, and limited supplier base of turn-key devices undoubtedly contributed to CRS’s undersized use but so too did the strong focus of the community on technical development over application. Perhaps tellingly, Olympus aborted the production of their CRS microscope only a few years after it was first launched.

In search of the next wave of success, a certain reckoning of the limitations of CRS imaging was inevitable. One of the shortcomings of common CRS microscopy implementations, which utilized narrow bandwidth lasers to drive a single Raman line, was that fast imaging only yielded information about a very narrow portion of the vibrational spectrum. Although orders of magnitude faster than spontaneous Raman microscopy, the narrow bandwidth version of nonlinear Raman imaging provided not nearly as much spectroscopic information as its linear counterpart. The transformation into a genuine chemical imaging tool requires the expansion of the spectral content without significantly compromising CRS’s speed advantage.^29^^–^^31^ Driven by this need, much research in the 2010s has been dedicated toward developing fast hyperspectral CRS imaging approaches.

Another limitation of CRS was its sensitivity. Although imaging of free-standing single lipid bilayers generated hope for biological applications early on,^32^ the number of molecules needed to produce such a signal were still in excess of $10^{5}$. Whereas fluorescence microscopy techniques can pick up signals that originate from single molecules, even the most sensitive CRS methods require tens of thousands of target molecules to produce a detectable signal. Consequently, the focus on improving sensitivity is an important strategy for broadening the use of the CRS technique.

The contrast in CRS imaging derives from the vibrational signatures of molecular compounds. For endogenous molecules, the number of such signatures is limited. The combination of a vast number of species and a limited number of vibrational signatures results in a dense spectrum of overlapping band structures. Identifying individual species against this background constitutes a fundamental challenge in vibrational spectroscopy. Therefore, methods that enhance the molecular specificity of CRS have the opportunity to expose the technique to a wider range of applications.

These three performance targets, hyperspectral imaging speed, sensitivity, and molecular specificity, have served as important motivations for the developments in the field of CRS microscopy during the past decade. In the following, we reflect on these targets and take stock of the current status of the technique’s capabilities.

## 3 Specificity

In the past decade, the development and use of Raman tags have grown significantly to identify specific metabolites. The general strategy of Raman tagging is that particular targets are equipped with chemical groups that have their characteristic vibrations in a frequency range free of overlapping band structures. Perhaps the gentlest form of Raman tagging is the substitution of a given element with one of its isotopes, which alters the effective mass of the mode and this shifts its vibrational energy. A prime example is the replacement of carbon-bonded hydrogen atoms with deuterons, which moves the mode vibration into the cell silent region of the spectrum. This concept was already employed to improve specificity of the world’s first CRS microscope^2^ and has proven early on a reliable strategy for identifying specific fatty acids in membranes.^33^^,^^34^

The need for Raman tags arose from the limited molecular information available from endogenous sources. Lipid imaging is so successful, in part, due to the isolated frequency range of the carbon–hydrogen (C–H) bond vibrations. In the absence of overlapping bands from other chemical group vibrations, the C–H stretching vibrations are confidently assigned. Their signal is used as a reliable source of contrast in CRS images. In direct contrast is the fingerprint region, known for its richness in information, but correspondingly, an intensely congested spectral range where individual bands’ assignment is immensely challenging due to their overlapping nature.

The molecular specificity of vibrational bands is limited, even when vibrational lines can be resolved and pinpointed. For instance, the amide I vibrational mode can often be discriminated from nearby vibrational lines, but targeting this mode provides information on all protein structures in the probed volume rather than a specific enzyme’s whereabouts. Similarly, the strong stretching mode of the carbon–carbon double bond (C–C) reports on the presence of numerous molecular species that contain a C–C moiety, complicating the identification of a specific target based on this band alone. Given these challenges, it is no surprise that the idea of Raman labeling has proved powerful in dramatically improving the molecular specificity of Raman-based imaging techniques.

The use of deuterated probes gained new momentum during the past decade through the realization that isotopic analogs of endogenous molecules can be metabolically converted in live cells and tissues, thus providing a “hook” to track metabolic processes. For instance, uptake and subsequent esterification of deuterated fatty acids or cholesterol offer a direct view of the cellular machinery a work.^35^^–^^37^ This principle can be extended to deuterated amino acids to follow protein synthesis *de novo*,^38^^,^^39^ deuterated glucose analogs to monitor lipogenesis,^40^^,^^41^ glycogen,^42^ and biomass production,^43^ as well as $D_{2}O$ to study protein and lipid metabolism in live animals.^44^ The ability to directly examine metabolic pathways in live biological systems offers a unique view of fundamental processes such as cellular homeostasis, tissue growth, aging, and oncogenesis. Hence, although the use of deuterated probes may appear a small technical feat, it is transformative in the way it has expanded the biomedical impacts of CRS microscopy.

Although the carbon–deuterium (C–D) bond offers a labeling strategy of minimal chemical interference, the Raman response from individual bonds is relatively weak, necessitating probes that incorporate numerous deuterons to achieve nominal CRS signals. Alternatively, molecular targets can be equipped with small chemical motifs that exhibit much higher Raman cross sections. The approach was popularized for CRS through the use of bio-orthogonal alkyne tags, small chemical groups with an exceptionally strong Raman response, and a vibrational resonance in the cell silent region of the spectrum.^45^^–^^47^ Alkyne tags are unreactive under typical physiological conditions and small enough to avoid compromised metabolism of its molecular carrier. These tags have been incorporated into a wide range of small molecules, enabling the visualization of specific nucleic acids,^46^^,^^47^ amino acids,^46^^,^^47^ glucose,^48^ fatty acids,^45^^,^^49^ sterols,^50^ choline,^46^ and many more, in live cells and tissues. This idea has been expanded to include other tags, including nitriles and isonitriles. Nitriles have the added benefit of a resonance frequency that is sensitive to the local electrostatics of its immediate chemical environment, allowing, for instance, local probing of water content.^51^

The use of Raman tags is not unique to CRS microscopy *per se*. In fact, the concept was introduced to improve the molecular specificity in conventional Raman microscopy, and many of the tag-bearing small molecules now used in CRS imaging were first developed for linear Raman applications.^52^^,^^53^ Nonetheless, the isolated nature of the spectral bands associated with these tags allows direct identification with narrowband CRS microscopy. Here the fast imaging capabilities of CRS imaging offer tangible benefits, as metabolic processes in live cells and organisms can be tracked in real time, a capability that is simply out of reach for spontaneous Raman microscopy.

## 4 Sensitivity

The sensitivity of CRS imaging is generally in the range of 1 to 10 mM for endogenous compounds. The use of Raman tags, which typically have higher Raman cross sections, can push the sensitivity into the ∼100-μM range. Although impressive, this detection limit is often higher than the concentration of small endogenous molecules and metabolites in the cell. The application space of CRS microscopy would thus widen significantly if ways can be found to improve the sensitivity.

Since the illumination power is limited by sample damage, the only conceivable way to improve the CRS sensitivity is to increase the effective Raman cross section of the molecular responders. For endogenous compounds, the Raman cross section is an inherent property of the molecule, which is essentially immutable to change when excitation wavelengths are far from electronic resonances. For extrinsic probes, however, Raman cross sections can be boosted significantly when electronic resonances are present near the frequency of the excitation beam. The principle of resonance Raman scattering can be ported to CRS excitation and correspondingly improve the molecular concentration detection limits.^54^^–^^56^ This strategy requires chromophores that exhibit vibrational modes with good couplings to the electronic resonance. Select fluorophores developed for the purpose of fluorescence labeling display excellent enhancement of the vibrational response of up to $10^{6}$ times when the excitation frequencies are tuned near the electronic transition, as demonstrated for stimulated Raman scattering (SRS).^55^^,^^56^ The result is that such fluorescent probes can be detected through the CRS process at sub-μM concentrations. This is significant, because it opens up the possibility to map distinct probes in a multi-label sample, with the number of different probes ultimately limited by the bandwidth of the Raman line rather than by the bandwidth of fluorescence. Whereas it is challenging to label the sample with more than four probes in fluorescence microscopy because of cross talk between the detection channels, in resonance-enhanced SRS imaging multiplex labeling can be extended to tens of different probes.^57^^,^^58^ In terms of multiplex imaging, this capability is a big win as many cell biological studies require the visualization of multiple molecular actors to uncover intracellular processes and pathways.

The multiplex capability offered through resonance-enhanced SRS can be pushed even further to lower probe concentrations. By letting the probe selectivity be determined by the SRS excitation process alone, the bandwidth on the detection side can, in principle, be relaxed. This implies that after a Raman transition, the molecule can be excited in a second step to an emitting electronic state, allowing efficient detection of the chromophore via fluorescence emission. If the electronic transition is conditional on a successful Raman transition, then the chromophore can be selected based on its narrow Raman band while the probing sensitivity is boosted by the highly efficient fluorescence detection strategy. This process, called stimulated Raman excited fluorescence (SREF), makes it possible to detect molecules at concentrations down to the single-molecule limit.^59^ The SREF method combines the advantages of multiplex imaging offered by SRS with the high sensitivity of fluorescence microscopy, enabling imaging studies that were hitherto impossible with either of these techniques alone.

## 5 Speed

The advances discussed in the previous two sections focused on CRS improvements through exploiting and optimizing the target molecule’s response. The intelligent use of molecular probes expands CRS’s application landscape without technical advancements of the microscope instrument itself. Next, we will focus on increasing the image acquisition speed of the microscope, an advance that often involves modifications of the system’s hardware. In particular, we are interested in ways that fast hyperspectral CRS imaging can be accomplished. The term “hyperspectral” is used here to denote the acquisition of data cubes where there are two spatial dimensions (typically the lateral coordinates x and y) and one spectral dimension, resulting in a 2D spatial image where each spatial pixel is expanded along the third coordinate to represent the vibrational spectrum.

Over the last decade, there has been a proliferation of approaches for recording CRS hyperspectral data cubes, many more than can be covered in this brief perspective. The variety of approaches is vast, which implies that there are also numerous ways to categorize them.^30^^,^^31^^,^^60^ For the discussion here, it will be helpful to categorize hyperspectral CRS techniques in terms of the detection strategy, namely either the use of a single pixel detector or the involvement of a detector array. For the case of a detector array, a spectral dispersion element is used, and numerous spectral values are recorded simultaneously. Where a single pixel detector is used, a data stream of values needs to be recorded in time from which spectral information can then derived.

By the early 2000s, it was recognized that spectral information in microscopic CARS measurements could be obtained efficiently by using a standard spectrometer as the detector.^61^^–^^63^ The speed of the resulting images is then limited primarily by the strength of the signal and the data processing time of the arrayed detector. Various optimization strategies have led to the acquisition of broadband CARS spectra, spanning the 600 to $3100\;{\mathrm{cm}}^{-1}$ range, with pixel dwell times as short as 3.5 ms for biological samples.^64^ For comparison, the fastest spontaneous Raman microscope requires at least 100 ms to obtain similar quality spectra.

The use of conventional arrayed detectors is more challenging in SRS microscopy because of the necessity of high-frequency modulation. One approach is the use of multiple lock-in detectors, each capturing the information from a spectral bin,^65^ a strategy that has been expanded to no less than 128 lock-in amplifiers.^66^ Other arrayed detectors used for SRS have included fast CMOS detectors^67^^,^^68^ and arrayed photodiodes where each element is equipped with a resonant LC filter.^69^ Using the latter recipe, SRS spectra consisting of 32 bins have been recorded within only 5  μs, corresponding to 200,000  spectra/s.^70^

Arrayed detectors offer limited flexibility in balancing the number of spectral data points with acquisition speed. For this reason, many CRS methods have focused on variations in the use of fast single-element detectors. The most straightforward method is the rapid spectral scanning of one of the input beams in narrowband CRS while recording images sequentially for each spectral setting.^71^^–^^73^ If starting from a broadband beam, a rapid tunable filter, such as a galvanometric mirror and a grating^74^ or acousto-optic tunable filter (AOTF),^75^ offers a viable way to collect CRS hyperspectral data cubes swiftly. Another popular approach is the spectral focusing method, used both for CARS^76^^–^^78^ and SRS,^79^^–^^81^ where the spectral dimension is obtained through a temporal scan of the delay between the chirped broadband pump and Stokes pulses. Rapid scanning of the delay is possible through various means, for instance with the aid of acousto-optic filters, producing spectral scans in as little as 12.5  μs per pixel.^82^ Other approaches use spectral modulation techniques, whereby the spectrum is amplitude modulated by a spatial light modulator,^83^ digital micromirror device,^84^ or AOTF,^85^ and the spectrum is retrieved computationally from a sequence of CRS measurements obtained for different realizations of the spectral content of the input beams. A similar concept can also be applied when a single-broadband beam is employed to drive the CRS process, using amplitude and phase shaping to achieve the desired resolution. Many spectral shaping techniques exist, most of them comprehensively summarized in Ref. 30.

A final development of note is found in the area of Fourier transform CRS, which rapidly collects temporal interferograms that can subsequently be transformed to the spectral domain to retrieve Raman-based spectra.^86^ Several optical designs for enabling fast sweeps in the temporal domain have made it possible to acquire spectra at very high rates. In point-scanning CARS microscopy, the Fourier transform approach has produced spectral acquisition times of 42  μs per spatial pixel.^87^ A related strategy has also been implemented in a wide-field CARS microscope.^88^ Outside of the microscope, even higher spectral acquisition rates have been reached,^89^ up to 100,000  spectra/s in the form of dual-comb coherent Raman spectroscopy.^90^^,^^91^

## 6 Taking Stock and Looking Ahead

The last decade’s technological developments are exciting, as they push CRS microscopy to its ultimate speed and sensitivity limits. Nonetheless, many implementations thus far remain rather complex, which has kept these solutions out of the hands of routine microscopy users. The success of an imaging technique like CRS is ultimately measured by the quality of the science and the discoveries it enables, and this requires the user base to expand beyond the community of microscope developers. Despite its impressive capabilities, the CRS microscope, in many ways, is still grappling with similar questions that bubbled up when the first CRS imaging system saw the light of day: how can this technology be tailored to benefit a much wider research community? Buttressed by a strong portfolio of biological applications in the scientific literature, CRS developers have put their hopes on commercial releases of the SRS microscope as a way to bring fast vibrational imaging properties to a larger group of users. An affordable and user-friendly SRS microscope would no doubt expand access to the technique and multiply the number of studies, in which CRS’s label free imaging capability is a decisive factor of success.

Beyond an emphasis on instrument development alone, the recent focus on Raman probe development offers another route out of the impasse. By expanding the uses of fast Raman-based imaging to include mapping of metabolic processes, multiplex probe microscopy and optimized Raman label strategies for visualizing key molecular actors, the technique is likely to reach new audiences in the decade ahead.
